# 

**Published:** 1908-04-25

**Authors:** 


Telegraphic Address: Ospedale, London.

THE MODERN MEDICAL JOURNAL.

Telephone Number: 2734 Gerrard.

LIBRARY
Indexed

NEW SERIES. No. 58, Vol. III.

No. 1130. Vol. XLIV.

SATURDAY, APRIL 25TH, 1908.

PRICE THREEPENCE.

